# An advanced computational intelligent framework to predict shear sonic velocity with application to mechanical rock classification

**DOI:** 10.1038/s41598-022-08864-z

**Published:** 2022-04-02

**Authors:** Majid Safaei-Farouji, Meysam Hasannezhad, Iman Rahimzadeh Kivi, Abdolhossein Hemmati-Sarapardeh

**Affiliations:** 1 School of Geology, College of Science, University of Tehran, Tehran, Iran; 2 Faculty of Petroleum and Natural Gas Engineering, Sahand University of Technology, Sahand New Town, Tabriz, Iran; 3 Institute of Environmental Assessment and Water Research, Spanish National Research Council (IDAEA-CSIC), Barcelona, Spain; 4 Associated Unit: Hydrogeology Group (UPC-CSIC), Barcelona, Spain; 5 Department of Petroleum Engineering, Shahid Bahonar University of Kerman, Kerman, Iran; 6 College of Construction Engineering, Jilin University, Changchun, 130600 China

**Keywords:** Solid Earth sciences, Energy science and technology, Engineering

## Abstract

Shear sonic wave velocity (Vs) has a wide variety of implications, from reservoir management and development to geomechanical and geophysical studies. In the current study, two approaches were adopted to predict shear sonic wave velocities (Vs) from several petrophysical well logs, including gamma ray (GR), density (RHOB), neutron (NPHI), and compressional sonic wave velocity (Vp). For this purpose, five intelligent models of random forest (RF), extra tree (ET), Gaussian process regression (GPR), and the integration of adaptive neuro fuzzy inference system (ANFIS) with differential evolution (DE) and imperialist competitive algorithm (ICA) optimizers were implemented. In the first approach, the target was estimated based only on Vp, and the second scenario predicted Vs from the integration of Vp, GR, RHOB, and NPHI inputs. In each scenario, 8061 data points belonging to an oilfield located in the southwest of Iran were investigated. The ET model showed a lower average absolute percent relative error (AAPRE) compared to other models for both approaches. Considering the first approach in which the Vp was the only input, the obtained AAPRE values for RF, ET, GPR, ANFIS + DE, and ANFIS + ICA models are 1.54%, 1.34%, 1.54%, 1.56%, and 1.57%, respectively. In the second scenario, the achieved AAPRE values for RF, ET, GPR, ANFIS + DE, and ANFIS + ICA models are 1.25%, 1.03%, 1.16%, 1.63%, and 1.49%, respectively. The Williams plot proved the validity of both one-input and four-inputs ET model. Regarding the ET model constructed based on only one variable,Williams plot interestingly showed that all 8061 data points are valid data. Also, the outcome of the Leverage approach for the ET model designed with four inputs highlighted that there are only 240 “out of leverage” data sets. In addition, only 169 data are suspected. Also, the sensitivity analysis results typified that the Vp has a higher effect on the target parameter (Vs) than other implemented inputs. Overall, the second scenario demonstrated more satisfactory Vs predictions due to the lower obtained errors of its developed models. Finally, the two ET models with the linear regression model, which is of high interest to the industry, were applied to diagnose candidate layers along the formation for hydraulic fracturing. While the linear regression model fails to accurately trace variations of rock properties, the intelligent models successfully detect brittle intervals consistent with field measurements.

## Introduction

An accurate characterization of underground formations is the key to achieve optimized recovery of geo-energies, particularly in oil and gas reservoirs. Compressional (V_p_) and shear (V_s_) sonic wave velocities, routinely obtained from seismic surveys and wireline logging, play a first-order role in reservoir evaluation under in-situ conditions. Sonic velocity measurements provide significant insights into formation pore pressure^1^, rock physical properties, including porosity, pore geometry, pore fluid, and mineralogical content^2–4^, as well as rock stiffness, strength, and brittleness of target strata^5^, with a wide range of applications from reservoir management and development^6^ to a variety of geomechanical, geotechnical and geophysical studies^7,8^. Therefore, in-situ measurements of compressional and shear velocities, frequently using full-waveform recordings, for example, Schlumberger Dipole Sonic Imaging tool (DSI), should be incorporated into the standard practice for reservoir evaluation. However, given the high cost of implementation, borehole conditions, and out-of-date logging tools, acoustic shear velocity measurements are commonly missing spatially across the field or even partially at some intervals along the wellbore. As a consequence, field-scale characterizations primarily require filling in this data shortcoming.

Rock acoustic properties can be directly measured on core specimens in the laboratory. Nevertheless, laboratory measurements are more costly and time-consuming. Furthermore, multiple variables, including pore pressure, temperature, in-situ stresses, pore fluid, saturation degree, and rock mass scale properties, come to influence the sonic wave propagation across the rock^2,9–11^. Replicating in-situ conditions in the laboratory may be challenging and introduce further uncertainties to the measurements. These experimental challenges have motivated researchers to develop shear velocity proxies from wireline logging data. Most notably, collecting velocity data from well logging, seismic, and laboratory measurements, Castagna et al.^12^ proposed a pioneering predictive model for shear velocity in siliciclastic rocks. They found an approximately linear relationship between shear and compressional velocities.

Castagna and Backus^13^ adapted the relation as a quadratic function for carbonate rocks. Since then, numerous empirical correlations have been proposed for shear velocity estimation in various rock types and saturated media, mainly in carbonate rocks, broadly encountered as hydrocarbon reservoirs^14–17^. Such empirical correlations are advantageous from an implementation point of view because compressional sonic velocity profiles are available in most wells. Eskandari et al.^18^ incorporated other conventional log suites of gamma-ray (GR), bulk density (RHOB), laterolog deep (LLD), and neutron porosity (NPHI) into a multivariate regression to deal with the potential effects of other environmental, fluid, and rock properties on shear velocity and promote the generalization capability of the models.

During the past two decades, artificial intelligence (AI) has drawn increasing attention in petroleum engineering and geosciences owing to its capability and robustness in modeling complicated phenomena, including reservoir fluid and rock properties^19–22^, hydrocarbon-bearing potential of source rocks^23^, rock failure behavior^24–28^, soil behavior^29,30^ and seismic characterization^31,32^. Predictive models thus got a boost with these new techniques. A great deal of research has also been dedicated to predicting shear velocity using a variety of artificial intelligent approaches^33,34^. Utilized intelligent models were found to contribute to more accurate velocity estimations. But how reliable are reservoir evaluations established upon the estimated shear wave velocities? Indeed, can small errors in velocity estimations give rise to dramatic discrepancies in estimates of physical, hydraulic, and mechanical properties of formations, which, in turn, may pose notable imperfections in engineering designs? How does the credibility of predictive models evolve with emerging new techniques and optimization schemes? And how do input variables control the model prediction capabilities? These are a number of key questions that are less well addressed and deserve a renewed investigation.

This study seeks the answer to the raised questions in the light of extensive modeling efforts in the context of a case study. The prediction of rock acoustic properties is brought to maturity by developing a large set of AI models. The data come from Sarvak limestone in a developing oilfield in the southwest of Iran. The models are built using wireline logging tracks along a wellbore. The employed models, whose governing algorithms are described in detail in the present study, consist of random forest (RF), extra tree (ET), Gaussian process regression (GPR), Adaptive neuro fuzzy inference system (ANFIS), and its optimization with differential evolution (DE) and imperialist competitive algorithm (ICA). The accuracy of the developed models is analyzed using different criteria. The synthesized velocity profiles are utilized to evaluate rock elastic properties and discuss how artificial intelligence can help improve the detection of candidate layers for hydraulic fracturing. The study manifests how untrustworthy are the linear shear velocity proxies for reservoir evaluation purposes.

## Data collection and processing

The candidate formation for this study is Sarvak carbonates of an oilfield in the southwest of Iran. The Sarvak formation, mainly composed of limestones, serves as a major oil-producing reservoir in this region. A variety of sedimentary features has been distinguished in Sarvak^35^, with the secondary porosity evaluated to range from 0 to 10% in the study area, implying a high degree of heterogeneity. The formation has an approximate thickness of 600 m, which is divided into upper and lower Sarvak layers separated by the 34-m-thick Ahmadi member. More than ten boreholes have been drilled to develop this reservoir, but only one well has full-waveform measurements registered by the Schlumberger DSI tool. Besides, conventional well logs, such as NPHI, RHOB, and GR, are available. The data set includes a total of 4048 data points, regularly recorded at depth intervals of 15.24 cm. Well logs are depth matched and then subjected to environmental and hole size corrections.

The lack of shear velocity measurements has posed significant challenges in conducting geomechanical studies in this area and motivated us to develop a robust predictive model. In the first step, the selection of input variables is of paramount significance. To this end, we seek physically sound relationships between shear velocity (Vs) as the output and other logging data as the inputs. Sonic velocities in carbonate rocks were found to depend primarily on mineralogy and, more importantly, the amount and type of porosity^36,37^. In formation evaluation, a combination of V_p_, GR, RHOB, and NPHI are frequently used for a detailed assessment of mineral contents and rock porosity. We establish two sets of predictive models: first, by using only V_p_ as the input parameter and then adopting the four well logs as the model variables, from now on referred to as one input and four inputs models, respectively. The reason for developing the former group is to find out how reliable these simple and widely used models are to directly bridge between compressional and shear velocities.

## Model development and performance assessment

### Modeling approaches

#### Gaussian process regression (GPR)

It was the late 1940s in which the Gaussian Process method was suggested and implemented for prediction purposes. This technique found its way into machine learning in the middle of the 1990s^38^. After that, numerous computer simulations tests were performed and confirmed the Gaussian Process (GP) method's high efficiency. One important positive point of Gaussian Process Regression (GPR) is its high power in processing multi-dimension, a limited number of samples, and non-linear difficulties^39^. Generally, a GP is a group of random variables in which a restricted number of these variables have a joint Gaussian scattering. A Gaussian Process (GP) is identified through a mean function and a positively defined covariance (kernel) function^40^.

Given a group of inputs $D=\left\{\left({x}_{i},{y}_{i}\right), i={1,2},\dots ,n\right\},$ ${x}_{i}\in$ R^d^, and ${y}_{i}$ $\in$ R.

The mean function is determined through:

1 $$m\left(x\right)=E[f\left(x\right)]$$

Covariance function is given by:

2 $$k\left(x,x{^{\prime}}\right)=E[(f\left(x\right)-m\left(x\right))(f\left(x{^{\prime}}\right)-m\left(x{^{\prime}}\right))]$$

In which: $x$, $x{^{\prime}}$ ϵ R^d^, and it is required to estimate $f(x*)$ for the testing data $x*$, after that, the GP could be given as:

3 $$f(x)\sim \mathrm{GP}[{\mathrm{m}}({\mathrm{x}}),{\mathrm{k}}({\mathrm{x}},{{\mathrm{x}}{^{\prime}}})]$$

Because of the regression type of difficulty, the model is defined as below^41^:

4 $$y=f\left(x\right)+\upxi$$

Affecting $\xi -N(0,{\sigma }_{y}^{2}$) subsequently, the previous distribution of observed value y is given.

5 $$y \sim N[0,K\left(X,X\right)+{\sigma }_{y}^{2}{I}_{n}]$$

The previous combination distribution of noted value y and estimated $f\left(x*\right)$ ^41^:

6 $$\left[\begin{array}{c}y\\ f*\end{array}\right]\sim {\mathrm{N}}\left(0,\left[\begin{array}{cc}0,K\left(X,X\right)+{\sigma }_{y}^{2}{I}_{n}& K(X,x*)\\ K(x*,X)& K(x*,x*)\end{array}\right]\right)$$

K (X, X) = K_n_ = K_ij_, it is n × n sequence positive definite matrix, the element of the K_ij_ = K (x_i_, x_j_) is implemented to calculate the correlation between x_i_ and x_j._ K (X,$$x*$$)=$${{\mathrm{K}}(x*,X)}^{-1}$$ is an n × 1 sequence covariance matrix between testing data $$x*$$ and training samples X. K ($$x*,x*)$$ shows the covariance of the test data; I_n_ represents n dimensions unit matrix^41^.

Accordingly, the posterior distribution of estimated value $f\left(x*\right)$ is achieved as below^41^:

7 $$P(f*\left|x*,X,y)\sim N(\mu *,\Sigma *)\right.$$

where:

8 $$\mu * =K(X,x*){[K\left(X,X\right)+{\sigma }_{y}^{2}{I}_{n}]}^{-1}y$$

9 $$\Sigma * =K\left(x*,x*\right)-K\left(X,x*\right){\left[K\left(X,X\right)+{\sigma }_{y}^{2}{I}_{n}\right]}^{-1}K(x*, X)$$

$\mu *$, $\Sigma *$ shows the mean and covariance of $f\left(x*\right).$

#### Kernel function

The key role of kernel or covariance functions in the Gaussian process is controlling GPR’s accuracy. The employed kernel function in the current study is automatic relevance determination (ARD) exponential.

#### Random forest (RF)

 RF is made up of a series of decision trees that are used to train trees concurrently. This method uses the efficiency of decision trees as the final choice for its model^42^.  The RF classifier's unique built-in feature selection attribute enables it to control a variety of input features without eliminating specific variables to minimize dimensionality^43^.

 The RF approach trains the classifier to use bootstrap aggregation (Bagging) to broaden the range of each tree in the forest. Markedly, the number of trees B is selected. B separates training data points from the core data according to this amount. Since bagging is viewed as an alternative for random sampling, around one-third of the database is unused to train each subtree. Any tree's residual data is known as the "out-of-bag" data point (OOB)^44^.

 In the RF method, due to the fact that the OOB may be applied to examine the model's efficiency by examining the OOB errors, cross-validation is not required ^45^.  For the training of any decision tree, it is mandatory to record the training sample for the tree. Suppose the training set as $D=\{\left({x}_{1}.{y}_{1}\right).\left({x}_{2}.{y}_{2}\right).\bullet \bullet \bullet \left({x}_{m}.{y}_{m}\right)\}$, ${D}_{t}$  highlights the training data for tree ${h}_{t}$, and ${H}^{oob}$  depicts the out-of-bag approximation result for sample x, thus ^45^:

10 $${H}^{oob}\left(x\right)=argmax{\sum }_{t=1}^{T}I({h}_{t}\left(x\right)=y$$

 And the error generalization for OOB data becomes:

11 $${\varepsilon }^{oob}\left(x\right)=\frac{1}{\left|D\right|}{\sum }_{(x.y)\epsilon D}I({H}^{oob}(x)\ne y)$$

 The randomness operation of the RF is controlled by the value K, which is typically specified as $k={log}_{2}d$ ^45^.  To determine the feature worth of each component X_i_, the factor is randomly quantized. The bellows value is used to quantify the relevance of a feature:

12 $$I\left({X}_{i}\right)=\frac{1}{B}\sum _{t}^{B}\widetilde{OOB}er{r}_{{t}^{i}}-OOBer{r}_{t}$$

 Here, ${X}_{i}$ denotes the permuted ith feature in the feature vector X, B suggests the percentage of trees in the RF, and $\widetilde{OOB}er{r}_{{t}^{i}}$  symbolizes the method forecast error for the perturbed OOB sample containing the permuted feature ${X}_{i}$ for tree $t$. $OOBer{r}_{t}$ refers to the original OOB data sample that contains the permuted component.

 The importance of the permutation feature signifies that an incredible importance quantity highlights that the feature is applicable in the estimation, and permuting the feature variable influences the model prediction. A minimal beneficial feature has no or little effect on the approximation of the system^46^. It should be noticed that the minimum leaf size and parent size for the constructed RF model were set to 1 and 19, respectively.

#### Extra tree (ET) 

 ET is a method of learning that applies an averaging strategy on Decision Tree projections in order to improve correctness and reduce processing complexity^47,48^.  The additional tree strategy generates a random set of trees. Their estimates are retrieved accurately, using arithmetic averaging in regression challenges and majority voting in classification issues. One significant distinction between the extra tree method and other tree-based machine learning algorithms is that neuron division occurs randomly via extra tree cut sites.

The trees are built in the opposite direction of a bootstrap replica, using the entire learning sample. In regression challenges, the procedure of extra tree splitting requires two key variables: (i) the frequency of random splits at each neuron, denoted by K, and (ii) the smallest size of the sample utilized to break a neuron, written by n_min_^47,48^*.*

 The additional tree algorithm grows trees by identifying the amount of K at each neuron and continuing this operation once leaves are reached. Unless all subsamples provide pure responses or the amount of learning samples is below n_min_^48^*.*  all subsamples produce pure responses. Extra trees are projected to adequately reduce variation by randomly assigning cut points and input features and by group averaging. Nonetheless, bias minimization can be accomplished by adding additional trees that utilize the complete original learning sample^47^.

In formal terms, provided a training data, $X=\{{x}_{1}.{x}_{2}.\dots .{x}_{N}$}, where the sample ${x}_{i}=\{{f}_{1}.{f}_{2.}\dots {f}_{D}\}$ ${f}_{j}$ as the feature and $j\epsilon \{1.2.\dots .D\}$.  Extra trees generate M unique DTs. In every DT, S_p_ indicates a portion of the training data X at child neuron p. Following that, the ETs algorithm selects the optimal split relating to S_p_ and a random segment of features for each neuron p^49^. It should be noted that the minimum leaf size and parent size for the developed ET model were set to 1 and 5, respectively.

#### Adaptive neuro fuzzy inference system (ANFIS)

 ANFIS, a widely used strategy for machine learning, combines neural networks with fuzzy systems. ANFIS's primary purpose is to alleviate the constraints of neural networks and fuzzy systems while maximizing the positive points of both methodologies.

 ANFIS utilizes the ANN learning procedure to derive rules from input and output data, resulting in the creation of a self-adaptive neural fuzzy system^50^. In general, three functions are available for building fuzzy systems: genfis1, genfis2, and genfis3^51^.  The genfis3 was used in the current report. The FIS framework is also constructed using a Sugeno system based on fuzzy C-means (FCM) clustering. Additionally, in fuzzy systems, membership functions may be chosen from a variety of functions^52^. In the current research, a Gaussian function was applied. The ANFIS and ANN training in this work were accomplished using a hybrid technique. This technique combines backpropagation and least-squares prediction. The input membership function elements are computed using backpropagation, while the output membership function factors are measured using the least-squares methodology.

**Figure 1:**
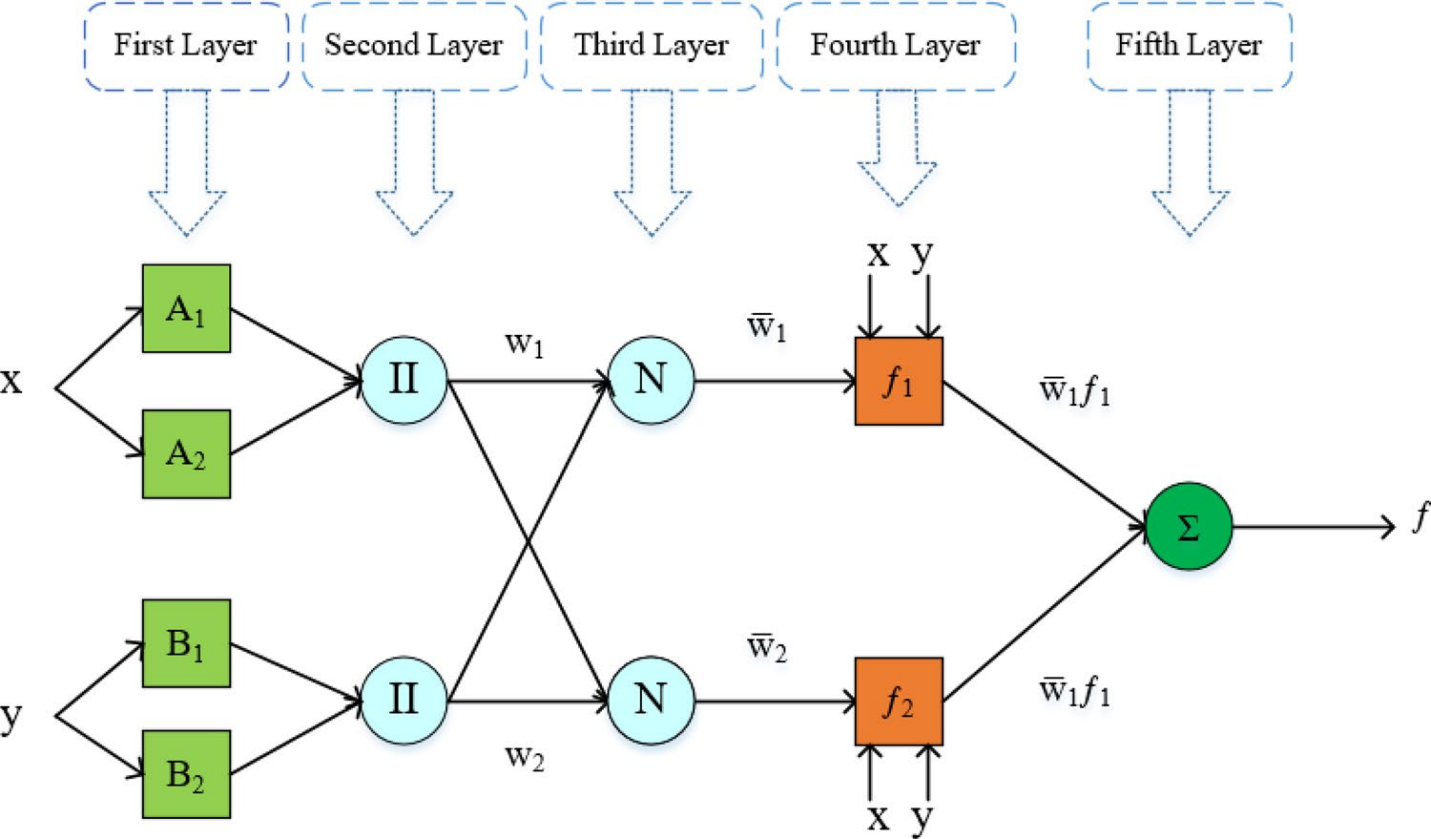
The schematic of the applied ANFIS model^19^

 ANFIS's architecture is composed of rules, input data, output membership functions, and membership degree functions. Fig. 1  illustrates the ANFIS design with two inputs. The first layer establishes each input's reliance on distant fuzzy areas. The next layer increases the weight of rules (*w*_*i*_) by raising the input numbers of each neuron. In the third step, the comparative weight of rules is determined. In the fourth stage, neurons are used to determine the contribution of rules to the output. The final layer, consisting a single neuron described as a stable neuron.^53^, is used to minimize the variance between the observed and forecasted output^54^.  As previously stated, the ANFIS paradigm is composed of five layers. The precise characteristics of each layer are listed below^55–58^. In this research, for the designed ANFIS model by one input, the number of nodes and fuzzy roles were defined 16 and 3, respectively. However, the number of nodes and fuzzy roles for the formed ANFIS model by four inputs were set to 57 and 5, respectively.

Layer 1:

 Layer 1 converts the incoming data to language terms. Each input criterion is associated with n neurons, each of which represents a preset linguistic phrase. The terms are produced in the initial layer in accordance with the previously specified membership functions. The Gaussian function used in this investigation is shown below:

13 $${O}_{i}^{1}=\beta \left(X\right)={\exp}\left(-\frac{1}{2}\frac{{\left(X-Z\right)}^{2}}{{\sigma }^{2}}\right)$$

$Z$ signifies the Gaussian membership function center in this calculation; $O$  denotes the output layer; and $\sigma$  reflects the variance term. The ANFIS program will optimize and alter these parameters during the learning period^59,60^.

Layer 2:

^59,60^:

14 $${O}_{i}^{2}={W}_{i}={\beta }_{Ai}\left(X\right).{\beta }_{Bi}\left(X\right)$$

Layer 3:

 In this layer, the firing energy of each rule is distinguished from the overall firing capacity of all rules by normalizing the recorded firing power parameters using the following equation^59,60^:

15 $${O}_{i}^{3}=\frac{{W}_{i}}{\sum_{i}{W}_{i}}$$

Layer 4:

 This layer recognizes the linguistic phrases associated with the model's output. The formula below determines the influence of each rule on the output of the model^59,60^:

16 $${O}_{i}^{4}=\overline{{W }_{i}}{f}_{i}=\overline{{W }_{i}}({m}_{i}{X}_{1}+{n}_{i}{X}_{2}+{r}_{i})$$

In this formula, ${r}_{i}$,${n}_{i}$, and ${m}_{i}$ denotes linear variables. The adjustment and optimization of these variables are performed through ANFIS  by the reduction of the discrepancy between predicted and target quantities^59,60^.

Layer 5:

 This layer use the weighted average summation technique to convert the complete collection of rules and an output to a numerical state according to the below calculation^59,60^:

17 $${O}_{i}^{5}=Y=\sum_{i}\overline{{W }_{i}}{f}_{i}=\overline{{W }_{i}}{f}_{1}+\overline{{W }_{2}}{f}_{2}=\frac{\sum_{i}{W}_{i}{f}_{i}}{\sum_{i}{W}_{i}}$$

### Optimization algorithms

#### Imperialist competitive algorithm (ICA)

ICA is a powerful technique based upon imperialism to expand the strength and law of a government far away from its geographical borders^61^. A first population starts this method as first countries—several best countries among the existing population regarded as the imperialists. Indeed, those countries with the minimum objective functions or costs, as an example, root mean square error (RMSE), are selected as the imperialists^62^.

The remaining population is considered as colonies and incorporated in the mentioned imperialists. After that, imperialistic competition starts between all the empires. Among the empires, the weakest one (with maximum RMSE) who is disabled to raise its strength and is disabled to succeed in the competition will be deleted from the competition. Thus, all colonies go toward their related imperialists associated with the competition between empires. In the end, hopefully, the mechanism of collapse will lead to reaching all the countries to a state where there is merely one empire around the globe (in the context of the issue), and all the other countries are colonies of that one empire. The most potent empire (with minimum RMSE) would be our remedy^63^.

#### Differential evolution (DE) optimizer

The DE optimizer is a swarm-based stochastic optimized defined by Storn and Price^64^. This practical algorithm has several merits: real coding, user-friendly, local searching feature, simplicity, and high speed^65,66^. The algorithm operates through the same computational processes employed by other evolutionary algorithms. The differential evolution algorithm utilizes the dissimilarity of the parameter vectors for exploring the objective space^67^.

### Statistical evaluation

To show and compare the constructed models, several parameters, namely average percent relative error (APRE%), average absolute percent relative error (AAPRE%), root mean square error (RMSE), and standard deviation (SD), were implemented. Formulas of these equations are provided below:

1. Average percent relative error (APRE):

18 $$E_{\rm r}=\frac{1 }{n} \sum_{i=1}^{n} (Ei)$$

In which $Ei$ is the relative deviation that is defined as:

19 $$Ei=\left[ \frac{Vs\left({\exp}\right)- Vs (cal) }{Vs (exp)} \right] \times 100, i=1, 2, 3,\dots ,n$$

2. Average absolute percent relative error (AAPRE):

20 $${\mathrm{AAPRE}}=\frac{1 }{n} \sum_{i=1}^{n} \left|Ei\right|$$

3. Standard deviation (SD):

21 $${\mathrm{SD}}=\sqrt{\frac{1}{n-1}\sum_{i=1}^{n}{ ( \frac{Vs \left(exp\right) - Vs \left(cal\right)}{Vs (exp)} )}^{2}}$$

4. Root mean square error (RMSE):

22 $${\mathrm{RMSE}}=\sqrt{\frac{1}{n} \sum_{i=1}^{n}{ ( Vs (exp) - Vs (cal) ) }^{2}}$$

In addition, the relevancy factor (r) was calculated to analyze the relationship between the inputs and outputs. The following formula was applied to calculate the relevancy factor (r) for input data:

23 $$r\left({input}_{k},output\right)=\frac{\sum_{i=1}^{n}({input}_{k,i}-{input}_{ave,k})({output}_{i}-{output}_{ave})}{\sqrt{{\sum }_{i=1}^{n}{({input}_{k,i}-{input}_{ave,k})}^{2}{\sum }_{i=1}^{n}{({output}_{i}-{output}_{ave})}^{2}}}$$

While *output*_*i*_ highlights the value of ith estimated output, *output*_*ave*_ implies the mean value of approximated output. *Input*_*k,i*_ displays the ith quantity of the kth input factor, while *Input*_*ave,k*_ displays the mean amount of the kth input variable^68^.

## Results and discussion

### Assessment of the validity and accuracy of one input-developed models

**Figure 2:**
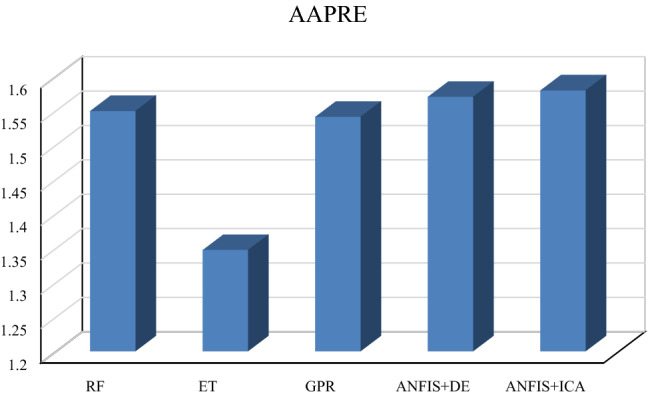
The AAPRE values of the five developed models based on one input (Vp).

**Figure 3:**
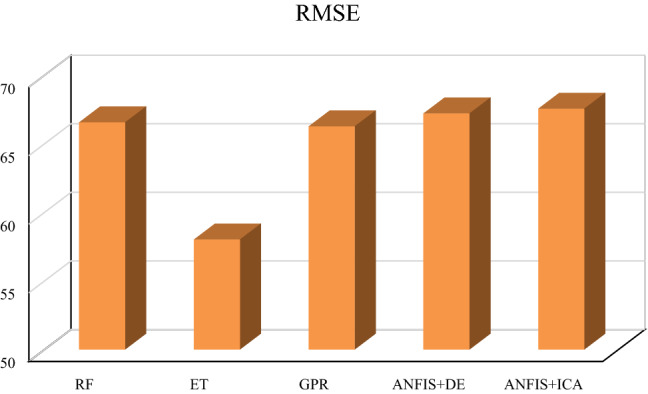
The RMSE values of the five constructed models based on one input (Vp).

Table 1 summarizes the obtained values of the parameters mentioned above for train, test, and total datasets in which one variable (V_p_) has been used as the input. As given in this table and Figs. 2 and 3, the smallest overall AAPRE (1.34%), RMSE (57.99), and standard deviation (0.019) belong to the extra tree (ET) model. After the extra tree model, the Gaussian process regression (GPR) indicates low values of overall AAPRE (1.54%) and RMSE (66.25). It is worth mentioning that the developed methods of Gaussian process regression (GPR) and random forest (RF) have closely similar AAPRE and RMSE values (Figs. 2 and 3). Likewise, a relatively similar performance for these two models can be concluded, based on the achieved values of AAPRE and RMSE. Collectively, the extra tree (ET) model can be regarded as the optimum model that estimated the target with substantially higher accuracy than those of the other models in the current study. The performance of the models based on the achieved error values can be summarized as below:

$${\text{ET}} > {\text{GPR}} > {\text{RF}} > {\text{ANFIS}} + {\text{DE}} > {\text{ANFIS}} + {\text{ICA}}$$

**Table 1:** The statistical parameters measured for one input models.

Model	RMSE (m/s)	AAPRE%	APRE%	SD
RF (Train)	67.10	1.56	−0.02	0.0222
RF (Test)	64.23	1.50	−0.04	0.0212
RF (All)	66.54	1.54	−0.43	0.0220
ET (Train)	55.23	1.29	−0.04	0.0183
ET (Test)	67.93	1.56	−0.04	0.0225
ET (All)	57.99	1.34	−0.04	0.0192
GPR (Train)	66.31	1.53	−0.04	0.0220
GPR (Test)	65.98	1.56	−0.06	0.0219
GPR (All)	66.25	1.54	−0.05	0.0220
ANFIS + DE (Train)	68.03	1.58	−0.06	0.0226
ANFIS + DE (Test)	63.70	1.50	0.03	0.0212
ANFIS + DE (All)	67.19	1.57	−0.07	0.0223
ANFIS + ICA (Train)	68.37	1.59	−0.06	0.0227
ANFIS + ICA (Test)	64.07	1.51	−0.12	0.0213
ANFIS + ICA (All)	67.54	1.58	−0.07	0.0224

**Figure 4:**
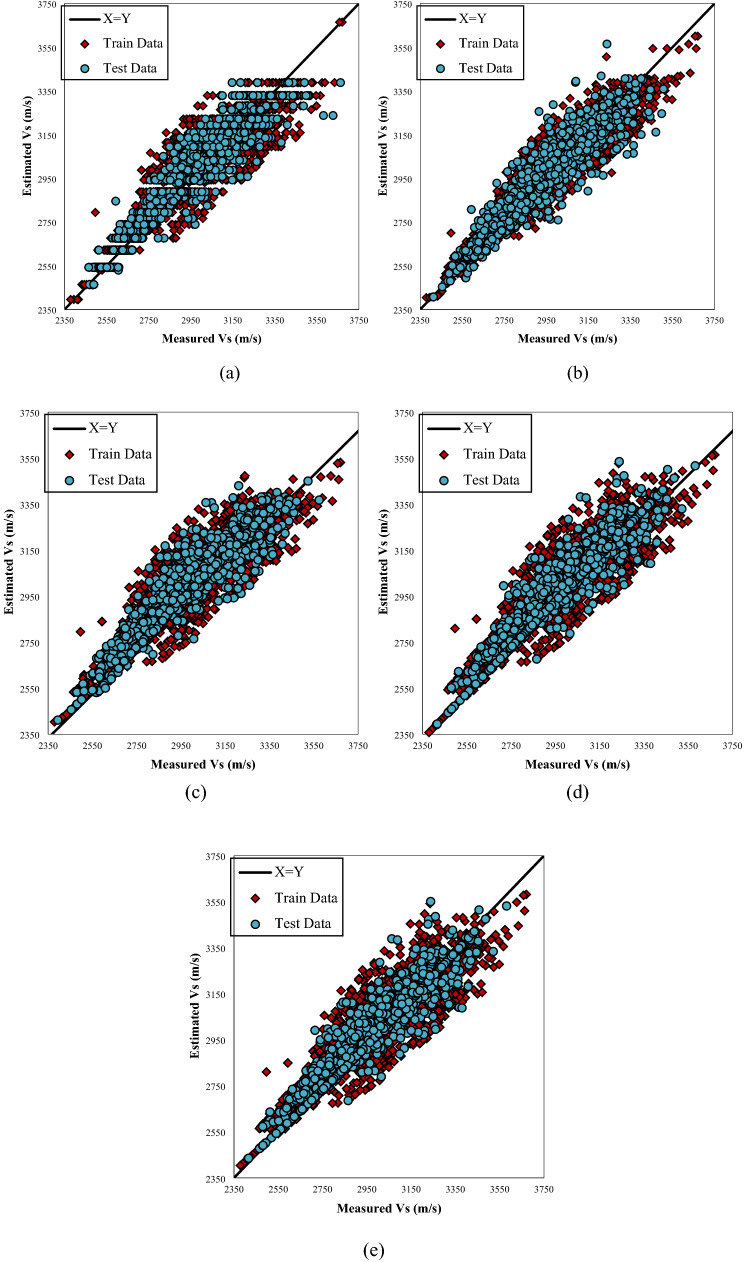
Plots of the developed paradigms (**a**) RF, (**b**) ET, (**c**) GPR, (**d**) ANFIS + DE, and (**e**) based on one input (Vp).

**Figure 5:**
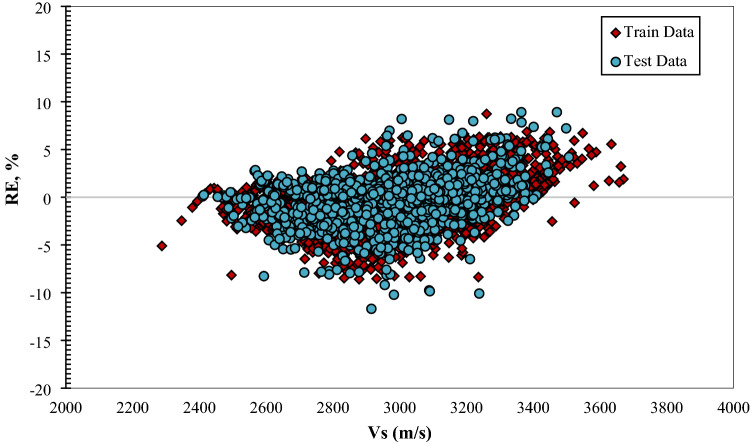
Relative error distribution of the designed ET model based on one input (Vp).

Figure 4  typifies the cross plots for the models utilized. The projected values are plotted versus the actual values in these illustrations. Cross plots exhibit the ideal model line by drawing the X=Y straight line amongst the experimental and approximation values. The closer the data on the plot are to the straight line, the better the model performs. As can be seen from these data, the predictions of the provided designs exhibit a high degree of consistency along the unit slope line. Fig. 5. depicts the error distribution profile for the developed extra tree (ET) structure, which is the optimal model. The system is more realistic in this picture if the errors are concentrated in a smaller zone close the zero-error line. Clearly, a substantial proportion of data is located near the zero line of the relative error (RE). This denotes the high accuracy of the developed extra tree (ET) model.

**Figure 6:**
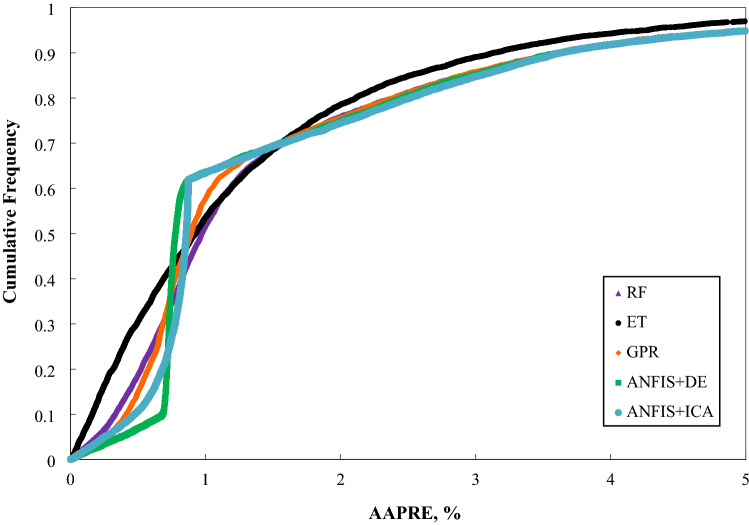
The cumulative frequency curve of the constructed models based on one input (Vp).

 Further, Fig. 6 depicts the cumulative frequency of the absolute relative error for the models used in this study. As indicated by this chart, the ET model is capable of approximating higher than 30% of Vs points with an absolute relative error of below 0.5 percent. Additionally, roughly 90% of the estimated Vs values through the ET model show an absolute relative error of lower than 3%. Correspondingly, The ET model's superior performance in predicting the Vs in contrast to other approaches can be deduced.

#### Outlier detection and utility domain of the constructed ET model (one input model)

 Outlier detection is a time-efficient method for finding a data set that is distinct from the rest of the data in a databank^69^.  The Leverage technique is a well-known methodology for detecting outliers, as it is based on data residuals (the departure of a model's expectations from experimental findings)^69–72^.  A hat matrix (H) is given in the leverage approach to establish the hat indexes or leverage of data as follows^69,73^:

24 $${\text{H}} = {\text{X}}\left( {{\text{X}}^{{\text{T}}} {\text{X}}} \right)^{ - 1} {\text{X}}^{{\text{T}}}$$

In which, X denotes a two-dimensional matrix containing N rows (data sets) and K columns (model features). Furthermore, T represents the transpose multiplier. The diagonal components of H typify the hat values of data^69,73^.

In a Williams plot, standardized residuals are plotted against hat values and various areas of out of leverage data, suspected data, and valid data are recognized. The standardized residuals’ formula (SR) for each data point is described as bellows^73^:

25 $$S{R}_{i}=\frac{{e}_{i}}{RMSE\sqrt{(1-{H}_{ii})}}$$

 In which ${e}_{i}$ represents the deviation of the estimated data from its experimental value (estimated output-measured data), RMSE stands for the root mean square error of the model, and ${H}_{ii}$ denotes the hat index of the *ith* data set.

In the leverage approach, warning leverage (H^*^) is determined to reject or accept the model results and calculations. This criterion is known as H^*^ $=\frac{3({k}+1)}{{N}}$ and commonly, a value of 3 with an SD of $\pm 3$ from the mean is selected to cover 99% of the dispersed data. Under the circumstances in which most of data sets end up within the intervals of $0\le {H}_{ii}\le$ H^*^ and $-3\le$ SR_i_ $\le 3$, it may be inferred that the proposed model and its approximations are valid, and the experimental data implemented for model development are reliable^69,73^.

**Figure 7:**
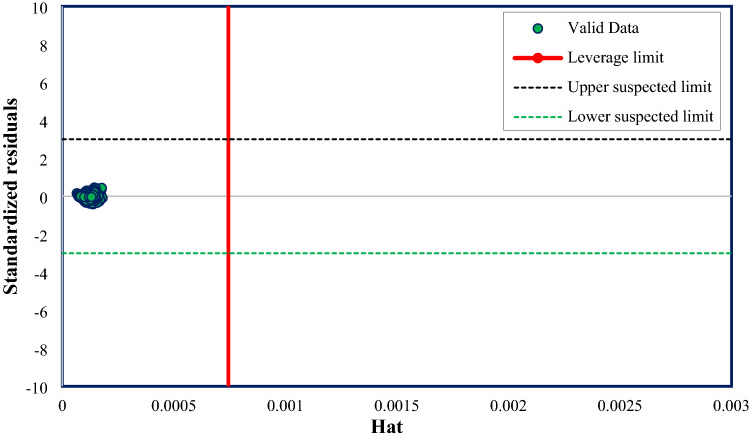
The Williams plot of the ET model based on one input (Vp).

The data points in the ranges of $-3\le$ SR $\le 3$ and H^*^ $\le H$ are known as good high leverage points. These points are outside the applicability area of the used model. The data sets that are situated in the interval of SR ˂ −3 or SR > 3 (notwithstanding their H value) are known as bad high leverage points. These data points are regarded as experimentally suspected data set that may be derived from an error over the experimental calculations^69,73^. Figure 7 depicts the Williams plot and notably implies that all 8061 data points are valid data.

#### Group error analysis (one input models)

**Figure 8:**
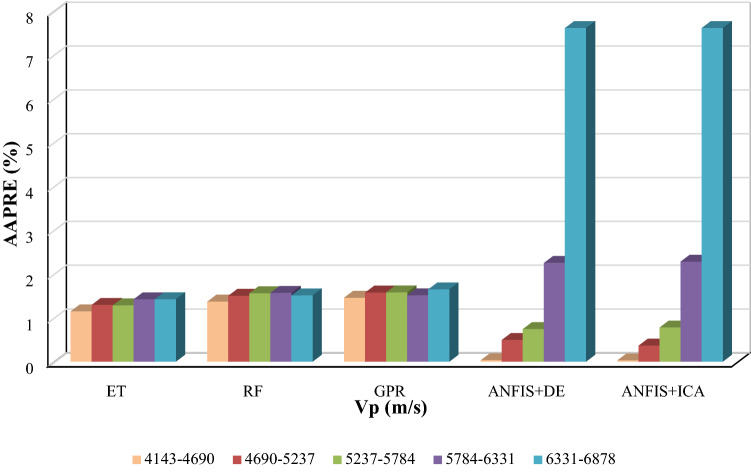
Group error diagram illustrating AAPRE values for the developed models based on one input (Vp).

In the group error technique, the error values along with the data in various intervals are measured and plotted. The data are separated into various intervals, and their error in each interval is measured and plotted^73^. In the present study, the Vp data points were divided into five sections, and the average AAPRE for each section was calculated. Figure 8 plots input values against AAPRE values for all five smart models. As can be observed, the extra tree (ET) model collectively provides lower AAPRE values compared to other models. However, it should be noted that although optimized ANFIS models demonstrate the higher AAPRE values in the range of 5784 to 6331 and 6331 to 6878 m/s than other models, the minimum AAPRE values within the first three sequences belong to these optimized models. Also, it is visible that for all five ranges of Vp values, the ANFIS model optimized with DE and ICA illustrates tightly similar trends.

### Assessment of the validity and accuracy of four inputs-constructed models

**Figure 9:**
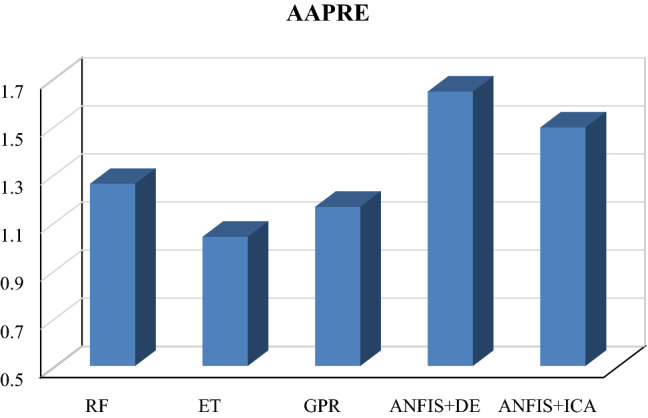
The acquired AAPRE% values for developed smart models based on four inputs.

**Figure 10:**
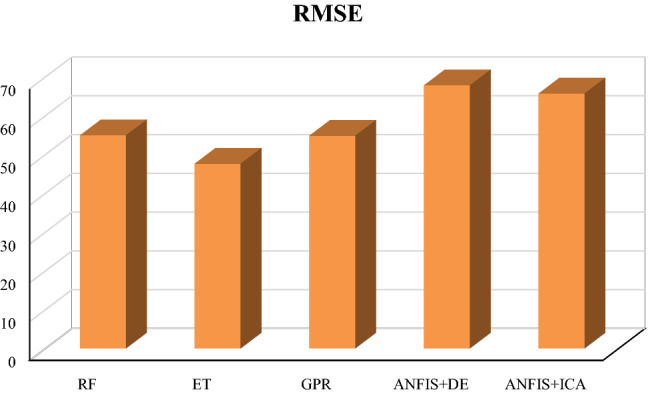
The obtained RMSE values for the developed models based on four inputs.

Table 2 summarizes the achieved values of the statistical parameters for train, test, and total datasets. As given in this table and Figs. 9 and 10, the extra tree (ET) model provides the smallest overall AAPRE (1.03%), RMSE (47.55), and standard deviation (0.015). The Gaussian process regression (GPR) ranked second based on the mentioned errors’ values. The GPR model indicates low values of overall AAPRE (1.16%) and RMSE (54.76). Similar to the constructed models based on one input, a closely similar performance can be concluded for the Gaussian process regression (GPR) and random forest (RF) models due to their subtle differences in acquired AAPRE and RMSE. Likewise, it can be noticed that the optimization algorithms’ performances do not differ considerably from each other. Therefore, the extra tree (ET) model can be recognized as the ideal model approximating the target (Vs) with higher accuracy than the other created models in this paper.

**Table 2:** The statistical parameters measured for four inputs models.

Model	RMSE(m/s)	AAPRE%	APRE%	SD
RF (Train)	53.45	1.22	−0.03	0.0177
RF (Test)	60.17	1.39	0.05	0.0198
RF (All)	54.86	1.25	−0.01	0.0182
ET (Train)	44.45	0.98	−0.03	0.0147
ET (Test)	58.29	1.25	−0.006	0.0192
ET (All)	47.55	1.03	−0.03	0.0157
GPR (Train)	52.84	1.12	−0.03	0.0174
GPR (Test)	61.84	1.28	−0.009	0.0205
GPR (All)	54.76	1.16	−0.02	0.0180
ANFIS + DE (Train)	67.33	1.6322	0.1053	0.0223
ANFIS + DE (Test)	69.39	1.6691	0.11	0.0230
ANFIS + DE (All)	67.74	1.6396	0.10	0.0224
ANFIS + ICA (Train)	65.16	1.4812	−0.07	0.0216
ANFIS + ICA (Test)	67.47	1.52	−0.03	0.0224
ANFIS + ICA (All)	65.63	1.49	−0.06	0.0217

The performance of the constructed models based on the acquired error values can be summarized as below:

$${\text{ET}} > {\text{GPR}} > {\text{RF}} > {\text{ANFIS}} + {\text{ICA}} > {\text{ANFIS}} + {\text{DE}}$$

Even though the performance of the models developed with one input follows the above trend except for optimizers, generally lower error values have been obtained when models are developed with four inputs.

**Figure 11:**
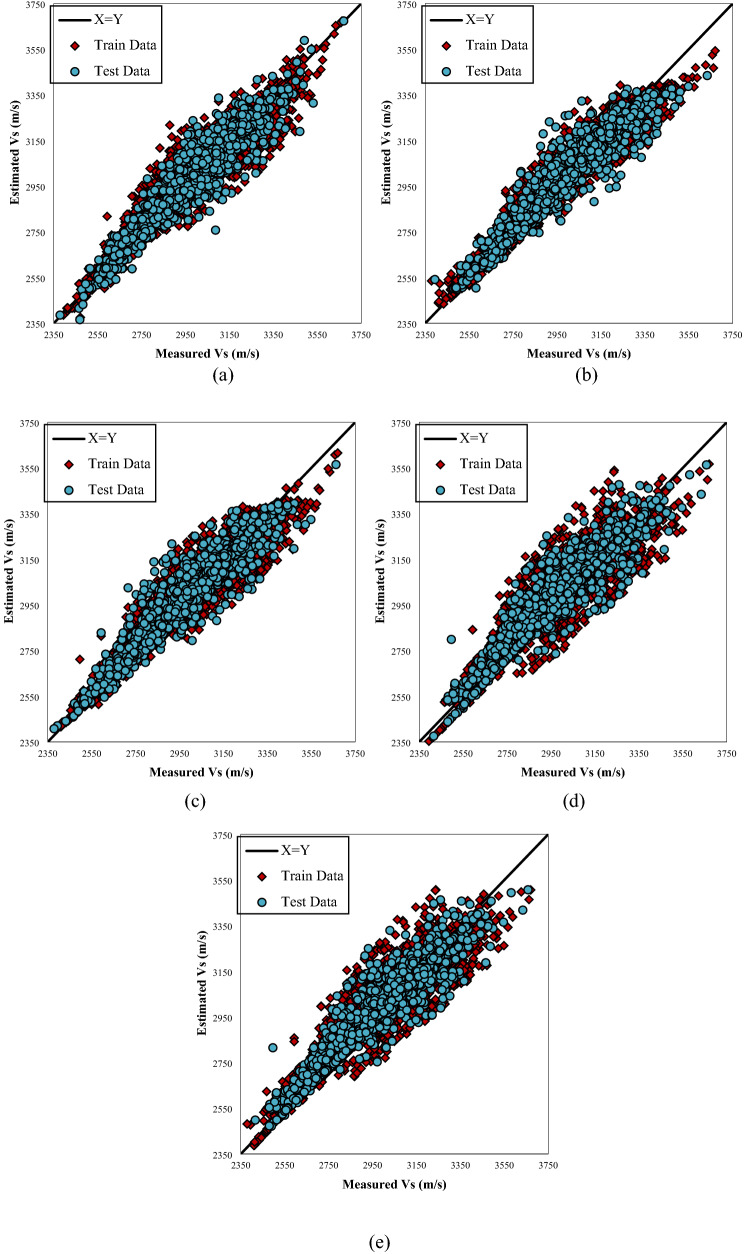
Cross plots of the developed models (**a**) RF, (**b**) ET, (**c**) GPR, (**d**) ANFIS + DE, and (**e**) ANFIS + ICA based on four inputs.

Figure 11 shows the plots of the applied systems. From these cross plots, it is apparent that the predictions of the applied models generally demonstrate a highly satisfactory agreement with the straight line. However, it can be observed that the data set belonging to the extra tree (ET) model (Fig. 11c) are closer to the unit slop line.

**Figure 12:**
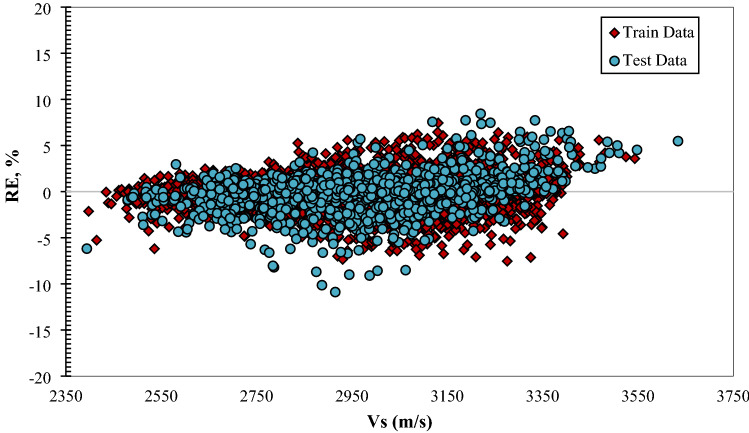
Relative error distribution curve for the extra tree (ET) model based on four inputs.

Illustrating the error distribution curve of the optimum model is another tool implemented to assess the developed models based on four inputs graphically. Figure 12 shows this curve for the developed extra tree (ET) as the ideal model. As it is visible, the major part of the data points has been situated near the zero line of the relative error (RE). This suggests the high accuracy of the developed extra tree (ET) model.

**Figure 13:**
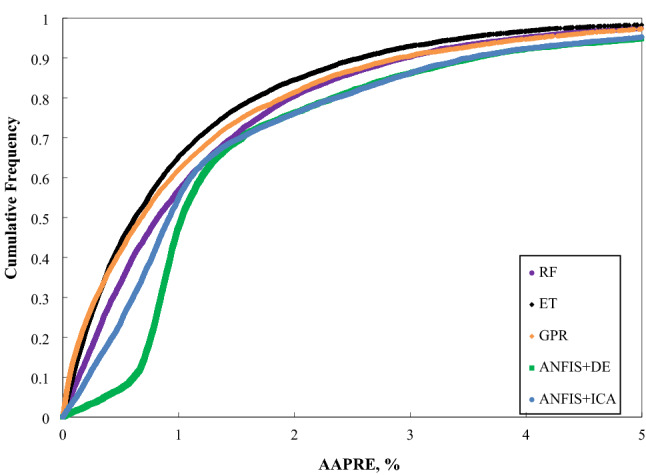
The cumulative frequency curve of the developed models based on four inputs.

The cumulative frequency of the models’ absolute relative error applied based on four inputs, and created correlation is depicted in Fig. 13. As this figure clarifies, the ET model could estimate approximately 93% of Vs points with an absolute relative error of less than 3%. Correspondingly,  the ET model's superior effectiveness in forecasting Vs than other strategies can be concluded.

#### Sensitivity analysis of the ET model (four inputs model)

**Figure 14:**
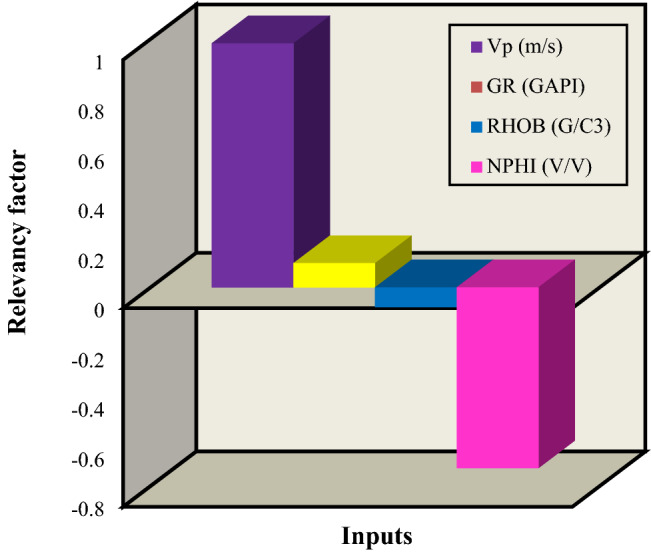
The relevancy factor diagram of four inputs.

Sensitivity analysis investigates the effect of a model’s input variation on the model’s output value. In this regard, the relevancy factor is a proper method. The relevancy factor calculates the amount of each input parameter influence on the output. A higher value of relevancy factor (r) for an input indicates a more prominent effect by that input on the output^73^. Figure 14 typifies the effect of four inputs on the Vs as the target parameter in this research. It implies that the Vp has a considerably more significant influence on the Vs value in comparison with the other three inputs. Therefore, the generally similar performance of the one-input and four-input developed models based on the obtained errors can justify the sensitivity analysis outcome, denoting Vp used as the only input in the first scenario of this paper impose a higher impact on the Vs as the target parameter.

#### Outlier detection and utility domain of the constructed ET model (four inputs model)

**Figure 15:**
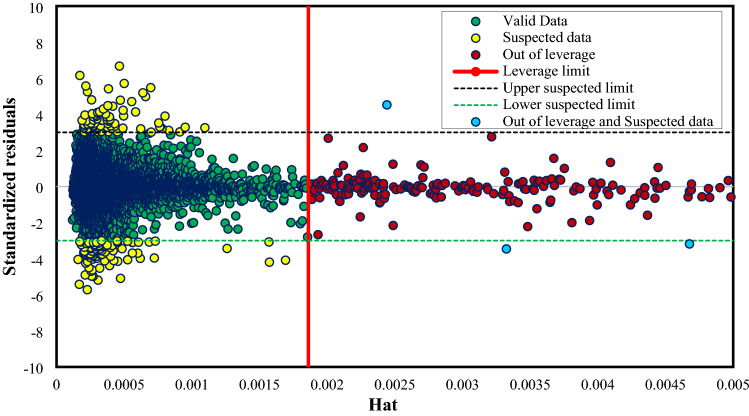
The Williams plot of the ET model with four inputs.

The result of the Leverage approach for the extra tree (ET) model constructed with four inputs is demonstrated in Fig. 15. It is plainly visible that most data sets are situated in the valid zone, and there are only 240 out of 8060 “out of leverage” data sets. Additionally, only 169 out of 8060 data points are suspected data. These amounts prove that the experimental data are reliable and that the developed ET model is statistically valid.

#### Group error analysis (four inputs models)

**Figure 16:**
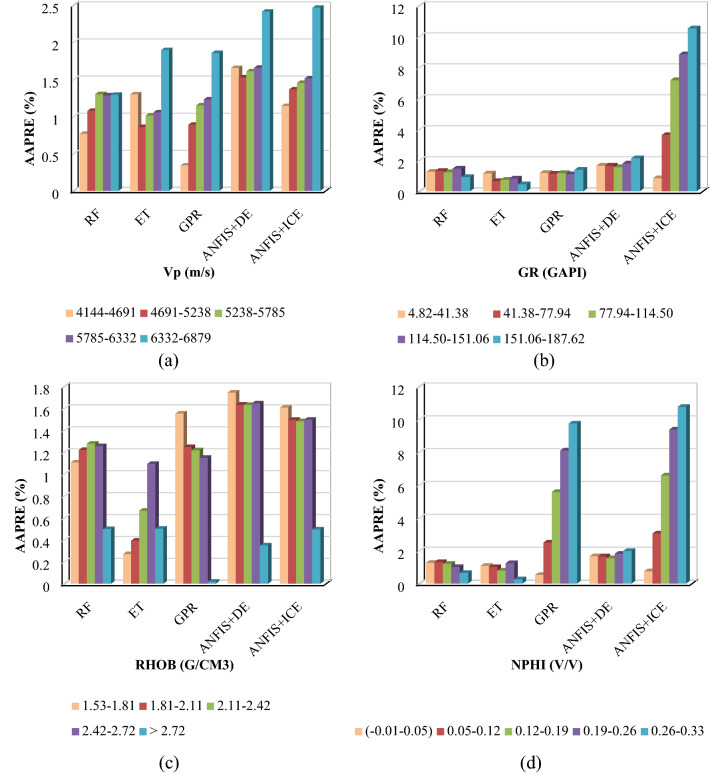
The group error distribution for inputs.

Figure 16 indicates the Group error distribution of four inputs within five divided sequences. For the Vp input case, as demonstrated in Fig. 16a, the smallest AAPRE within the interval of 4144 to 4691 belongs to the GPR model. The extra tree (ET) model shows a lower AAPRE than that of other developed methods for the other three ranges. Ultimately, in the range of 6332 to 6879, the random forest (RF) model indicates a lower AAPRE compared to those of other models.

In the case of the second input (GR) (Fig. 16b), it is evident that for all five defined intervals, the extra tree (ET) model has the minimum AAPRE. Regarding RHOB (Fig. 16c), the extra tree (ET) model generally typifies the lower AAPRE compared to other models. However, for the last defined range of RHOB values (> 2.72), the Gaussian process regression (GPR) model implies a notable lower AAPRE than that of other models. Finally, considering the NPHI input (Fig. 16d), the extra tree (ET) model collectively shows lower AAPRE values than other developed intelligent models.

### Implications to candidate selection for hydraulic fracturing

Hydraulic fracturing widely serves as an essential technique to enhance the productivity of low-permeability hydrocarbon reservoirs. Massive hydraulic fracturing involves the injection of large volumes of water at high pressure and rates, making economic production from gas shales of nano-darcy-range permeability viable^74,75^. However, not all depth intervals in the reservoir are appropriate for fracturing. Indeed, a promising selection of candidate layers for a fracturing completion is the key to ensure high profitability. The degree to which the rock is efficiently fractured to create a wide and sufficiently permeable fracture network for the hydrocarbon to flow is characterized by the brittleness index, BI^76,77^. Consequently, the literature has witnessed in recent years tremendous efforts to develop accurate and credible brittleness models (see, for instance, Kivi et al.^78^, Meng et al.^79^ for a review). Among them, Rickman et al.^75^ proposed a brittleness index $BI [-]$ by hypothesizing that brittle rocks possess relatively high Young´s modulus $E [Pa]$ and low Poisson´s ratio $\nu [-]$, which is as follows

26 $$BI=\frac{1}{2}\left(\frac{E-{E}_{min}}{{E}_{max}-{E}_{min}}+\frac{{\nu }_{max}-\nu }{{\nu }_{max}-{\nu }_{min}}\right)$$

where the superscripts “*min*” and “*max*”, respectively, stand for the least and highest elastic moduli values. The so-called elastic brittleness index has drawn widespread attention in field applications owing mainly to its simplicity and proficiency, proven through comparison with rock failure behavior in laboratory^17^ and field observations^80,81^. Elastic moduli can be conveniently evaluated from wireline logging data, which is written as:

27 $$E=\frac{\rho {V}_{s}^{2}\left(3{V}_{p}^{2}-4{V}_{s}^{2}\right)}{{V}_{p}^{2}-{V}_{s}^{2}}$$

28 $$\nu =\frac{{V}_{p}^{2}-2{V}_{s}^{2}}{2({V}_{p}^{2}-{V}_{s}^{2})}$$

where $\rho [kg/{m}^{3}]$, ${V}_{p} [m/s]$ and ${V}_{s} [m/s]$ denote the rock´s bulk density and compressional and shear sonic velocities, respectively. Equations (23) to (25) point to the importance of developing shear velocity proxies in optimizing the hydraulic fracturing operation where full-waveform sonic data are partially or thoroughly missing. To further highlight this significance, we evaluate the elastic moduli and brittleness profiles across the studied formation using the most accurate artificial intelligence models established in Sects. 4.1 and 4.2, i.e., ET models with one and four input variables, as well as linear regression model, which is of interest due to its simplicity to the industry. The extracted linear relation between the shear and compressional velocities is as follows:

29 $${V}_{s}=0.476{V}_{p}+268.03$$

where the velocities are in *m/s*. The developed correlation represents a high accuracy, characterized by an AAPRE and RMSE of 2.2 and 89.03, respectively, which are comparable to the values achieved from artificial intelligence models (see Tables 1 and 2). The resultant statistics seem to attest to the high precision of the constructed linear model.

**Figure 17:**
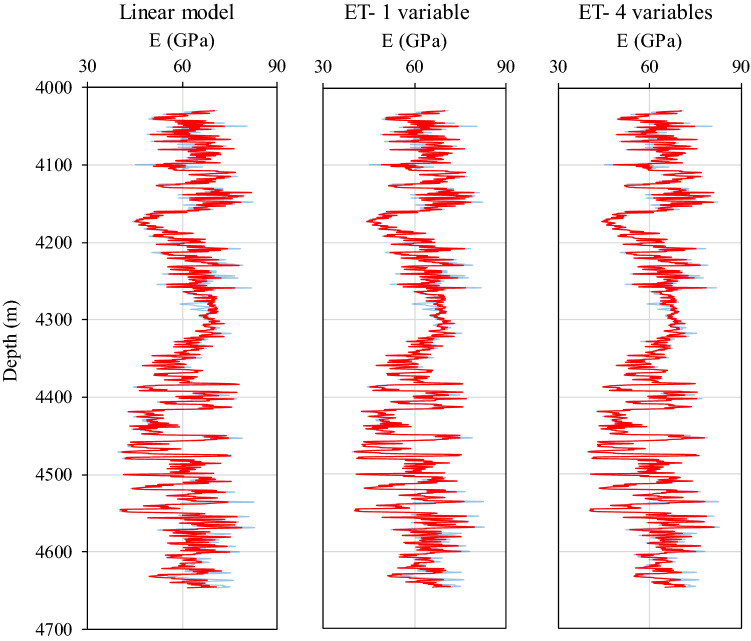
Vertical distributions of Young´s modulus along the formation. Calculations based on modeled shear velocities are illustrated in red, while measurements based on DSI data are included as blue curves on the background for the sake of comparison.

The reliability of the created models can also be inferred from the estimated profiles of Young´s modulus along with the examined formation (Fig. 17). The measured Young´s modulus tracks using modeled shear velocities (ET models and linear regression) and DSI data return almost a perfect match. However, discrepancies arise when comparing vertical distributions of Poisson´s ratio obtained from the mentioned three models (Fig. 17). The four-variable ET model estimates of shear velocity result in a Poisson´s ratio profile that is in good agreement with the actual one, i.e., calculated from DSI data. Although the single input ET model satisfactorily captures the general evolution trends of the Poisson´s ratio across the layer, a perfect quantitative match is missing. This comparison clearly highlights a key and complex dependence of sonic velocity on a set of contributing factors, which a combination of well logging data can only realistically reflect this complexity. This inconsistency may not necessarily pose major uncertainties to our analysis because what matters in candidate selection for hydraulic fracturing is the relative sequence of brittleness and not its absolute value.

**Figure 18:**
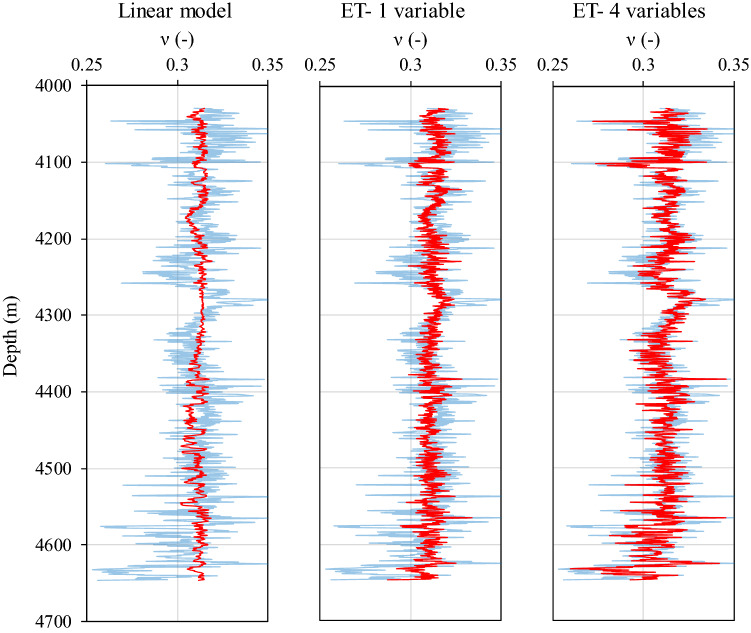
Vertical distributions of Poisson´s ratio along the formation. Calculations based on modeled shear velocities are illustrated in red, while measurements based on DSI data are included as blue curves on the background for comparison.

Interestingly, despite admissible velocity measurement capabilities, the linear model completely fails to estimate Poisson´s ratio profile neither qualitatively nor quantitatively. This disagreement is evidently due to the fact that Poisson´s ratio only depends on the ratio of compressional to shear velocity (see Eq. 25). Accordingly, the smaller the absolute value of the velocity intercept, the smaller the variability of Poisson´s ratio. Thus, the closer its value is to a constant controlled by the velocity ratio. The derived linear relation here narrows down the variation of Poisson´s ratio to as small as 0.3 to 0.32 (Fig. 18).

**Figure 19:**
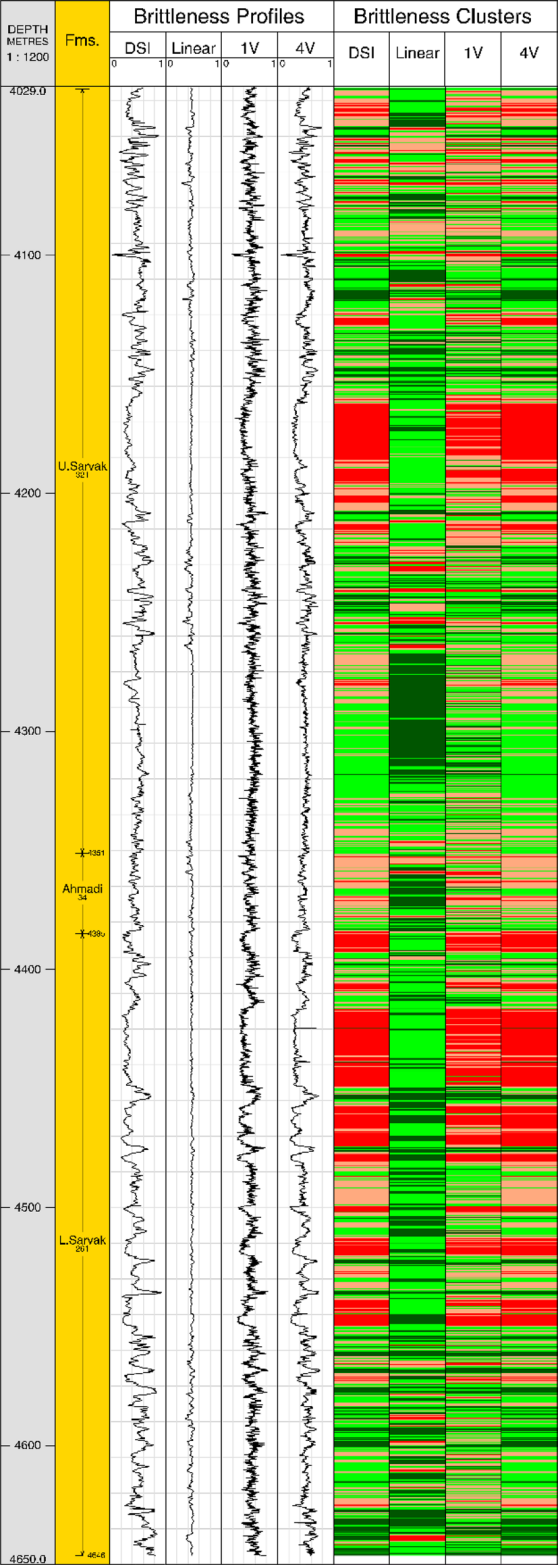
Brittleness-based rock clustering of Sarvak formation. The first four tracks depict the extracted brittleness profiles, distinguished by the source shear velocity, and what come next are the corresponding identified rock classes. The clusters are represented by red, purple, light, and dark green colors corresponding to an increasing order of brittleness.

We assessed the brittleness profiles along the formation using equation (33) and the predicted elastic moduli. We also employed the well-established k-means clustering technique^82^ to develop a mechanical rock classification and diagnose rock classes of different brittleness ranges. After a trial-and-error procedure for clustering, we assumed four rock clusters for illustration purposes. One should bear in mind that the number of rock clusters should be determined based on the identified rock types through a detailed geological analysis of recovered cores and thin sections^83^. Furthermore, for a robust screening of sweet spots, the clustering should also take into account other affecting parameters such as rock porosity, permeability, saturation, and in-situ stresses, which is out of the scope of this study. As expected from elastic moduli predictions, a comparison of brittleness profiles and clusters associated with ET model evaluations and recorded velocities discloses a good agreement (Fig. 19). The lowermost 100 m of the formation and some scattered intervals in its middle and top (light and dark green clusters) are found to have relatively higher brittleness compared to the adjacent zones (purple and red clusters). Therefore, the former groups can be considered as target layers for hydraulic fracturing while the latter potentially act as fracture barriers. However, the regression-based brittleness estimate, inheriting errors from elastic parameter calculations, is not able to follow the overall trends, and the associated fracturing design would be misleading. Briefly, it can be concluded that using linear models to estimate the shear sonic velocity gives rise to certain uncertainties in evaluating the rock Poisson´s ratio and negatively impacts subsequent geo-mechanical studies. Hence, their application to fill in data gaps should be restricted or treated cautiously. Instead, the employed intelligent approaches provide powerful tools for velocity estimations and should be taken as common practice in the industry.

## Conclusions

In this paper, two scenarios were adopted to estimate Vs from petrophysical well logs of GR, RHOB, NPHI, and Vp. For this objective, five different intelligent models of random forest (RF), extra tree (ET), Gaussian process regression (GPR), and the optimization of ANFIS with differential evolution (DE) and imperialist competitive algorithm (ICA) were employed.

In the first scenario, the target was predicted based on only Vp and the extra tree (ET) model provided lower AAPRE than other intelligent models. Furthermore, cross plotting the approximated Vp against its measured values for the extra tree (ET) model showed more uniformity than other implemented models. The error distribution curve also typified the high accuracy of the extra tree (ET) model. The cumulative frequency of the absolute relative error further supported better performance of the extra tree (ET) model than that of other developed models. Notably, the Williams plot of the data sets illustrated that all 8061 data point are valid. Ultimately, the group error analysis proved that the extra tree (ET) developed model has a lower AAPRE within all divided data sets than other models.

The second scenario predicted Vs from the integration of Vp, GR, RHOB, and NPHI inputs. Like the first approach, the minimum AAPRE was acquired by the extra tree (ET) model in this approach. Likewise, the cross plot of experimental Vp values versus its approximated values through the ET constructed model indicated more uniformity than other models. More acceptable performance for the ET model was demonstrated by its error distribution curve and cumulative frequency of the absolute relative error. The leverage approach also suggested that both measured data and the developed ET model are statistically valid. Also, the sensitivity analysis outcome denoted that the Vp has a higher impact on the target parameter (Vs) than other used inputs. Generally, it can be concluded that the second approach is more acceptable because of the lower achieved errors of its constructed models.

The field applicability of ET models as the most accurate developed intelligent approach was verified and compared with the linear regression model. The ET models, particularly that of the second scenario, satisfactorily estimated elastic moduli profiles in close quantitative agreement with field measurements and diagnosed brittle layers for hydraulic fracturing along Sarvak formation. Interestingly, although of acceptable accuracy, the regression-based velocity profile led to pronounced uncertainties in evaluating the rock Poisson´s ratio and subsequent geo-mechanical evaluations, for instance, as discussed in this study, the relative sequence of brittle layers for hydraulic fracturing. This highlights the outperformance of the established intelligent frameworks for sonic velocity estimations and strongly suggests their wide employment in reservoir evaluation practices. Nevertheless, the choice of appropriate input well-logging variables, particularly when any of the conventional log data is not available, and a universal intelligent model for estimating shear sonic velocity for different rock types yet remain a topic of ongoing research.
